# Improving Precise CRISPR Genome Editing by Small Molecules: Is there a Magic Potion?

**DOI:** 10.3390/cells9051318

**Published:** 2020-05-25

**Authors:** Nadja Bischoff, Sandra Wimberger, Marcello Maresca, Cord Brakebusch

**Affiliations:** 1 Biotech Research and Innovation Centre (BRIC), University of Copenhagen, Ole Maaløes Vej 5, 2200 Copenhagen, Denmark; nadja.bischoff@bric.ku.dk; 2 Discovery Sciences, BioPharmaceuticals R&D, AstraZeneca, Gothenburg, Pepparedsleden 1, 43 183 Mölndal, Sweden; sandra.wimberger@astrazeneca.com

**Keywords:** CRISPR efficiency, low molecular weight compounds, homology directed repair

## Abstract

Clustered Regularly Interspaced Short Palindromic Repeats (CRISPR) genome editing has become a standard method in molecular biology, for the establishment of genetically modified cellular and animal models, for the identification and validation of drug targets in animals, and is heavily tested for use in gene therapy of humans. While the efficiency of CRISPR mediated gene targeting is much higher than of classical targeted mutagenesis, the efficiency of CRISPR genome editing to introduce defined changes into the genome is still low. Overcoming this problem will have a great impact on the use of CRISPR genome editing in academic and industrial research and the clinic. This review will present efforts to achieve this goal by small molecules, which modify the DNA repair mechanisms to facilitate the precise alteration of the genome.

## 1. Introduction

The ability to manipulate DNA sequences by molecular biology techniques had a fundamental impact on experimental biology. Furthermore, the work of Herbert Boyer and Stanley Cohen on restriction enzymes paved the way for the establishment of Biotechnology Industries [1].

The most commonly used tools in DNA manipulation, including restriction enzymes and recombinases, are derived from defense mechanisms that prokaryotes have developed to fight viral infections, or from viral mechanisms of replication and survival to host defense mechanisms. Therefore, it is not surprising that the development of tools for *in cellular* DNA engineering of prokaryotes and eukaryotes has exploited similar bacterial or viral mechanisms.

The lambda Red recombination system from the bacteriophage lambda is currently the most efficient system of DNA engineering in prokaryotes [2]. This system exploits the ability of Redβ to anneal a single strand DNA donor to the single-strand DNA exposed during bacterial or plasmid DNA replication. The lambda Red recombination system is very dependent on the DNA replication status of the target locus, the electroporation, the stability of the incoming DNA donor and often requires the antibiotic selection to isolate a recombination event. A similar mechanism based on endogenous DNA annealing at the replication fork or the transcription bubble has been developed in lower eukaryotes such as the yeast *Saccharomyces cerevisiae* allowing efficient DNA engineering of these organisms. Unfortunately, higher eukaryotes are not prone to DNA manipulation by DNA annealing, probably due to their chromatin structure and their DNA repair system. Interestingly, Clustered Regularly Interspaced Short Palindromic Repeats (CRISPR) defense mechanisms exploiting Cas9 endonucleases and targeting RNAs are not naturally recombinogenic in bacteria and are not good tools for DNA engineering in bacterial cells without providing exogenous recombination systems. This is in contrast to the extended use of CRISPR/Cas9 derived tools for DNA engineering in eukaryotes. Most of the CRISPR/Cas9 tools are not directly inserting the desired modification but they are just generating repair intermediates like DNA Double-Strand Breaks (DSBs) or single-strand nicks, that promote exogenous DNA capture or random insertions or deletions (indels).

Thus, after introducing a CRISPR/Cas9-targeted DSB, which can be highly toxic to cells if not repaired, the cell’s DNA repair machinery is activated to join the loose DNA ends and determines the outcome of an editing event. There are two major repair categories: Homology Directed Repair (HDR) and End-Joining (EJ). The latter can be further divided into Non-Homologous End-Joining (NHEJ) and alternative End-Joining (a-EJ).

**Figure 1:**
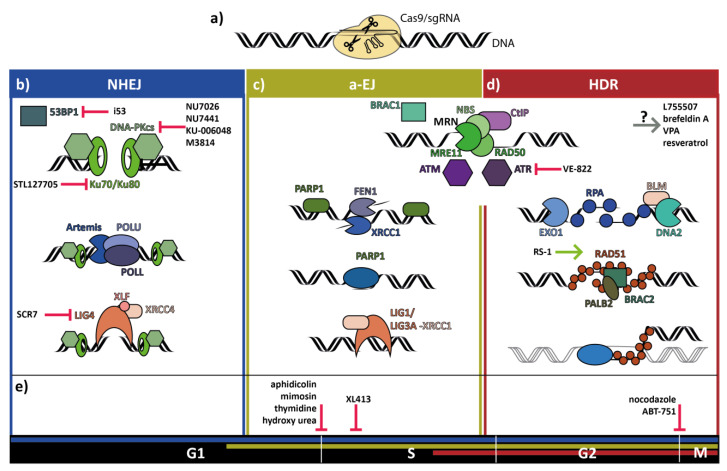
Major mammalian DNA damage repair pathways at Cas9-induced DSBs together with small molecules and one peptide (i53) reported to increase knock-in efficiencies. Shown are the three major repair pathways after a CRISPR/Cas9-induced DNA double-strand break. (**a**) Depicted is a Cas9/sgRNA complex cleaving DNA. (**b**) During Non-Homologous End-Joining (NHEJ) Ku70/Ku80 protect free DNA-ends from end resection. DNA-Protein-Kinase catalytical subunit (DNA-PKcs) phosphorylates different DNA repair enzymes. Ends are processed through Artemis, Polymerase Mu (POLM) and Polymerase Lambda (POLL) and ligated by the Ligase IV, X-Ray Repair Cross-Complementing Protein and 4 XRCC4-like Factor (LIG4-XRCC4-XLF) complex. (**c**,**d**) Breast Cancer Type 1 (BRCA1) antagonizes p53-Binding Protein 1 (53BP1) and enables end resection mediated by CtBP-interacting protein (CtIP) and the MRN complex Meiotic Recombination 11 (MRE11), RAD50, and Nijmegen Breakage Syndrome 1 (NBS1) necessary for alternative End-Joining (a-EJ) and Homology Directed Repair (HDR). The Kinases Ataxia Telangiectasia Mutated (ATM) and ATM-Rad3- related (ATR) function as damage sensors and activate different repair enzymes. (**c**) In a-EJ extensive end resection is prevented through Poly [ADP-ribose] Polymerase 1 (PARP1). After annealing of short homologies, X-Ray Repair Cross Complementing 1 (XRCC1) or Flap Endonuclease 1 (FEN1) cleave 5′-flaps and Polymerase Theta (POLQ) performs gap-fillings. Ligase I (LIG1) or Ligase III alpha-XRCC1 (LIGA-XRCC1) ligate DNA ends. (d) HDR requires extensive end resection mediated by Exonuclease 1 (EXO1) or Bloom Helicase and DNA2 Helicase/Nuclease (BLM-DNA2). Replication Protein A (RPA) binding of single-stranded DNA prevents the formation of secondary structures. RPA is replaced by RAD51 with the help of Breast Cancer type 2 (BRCA2) and Partner and Localizer of BRCA2 (BRAC2-PALB2). RAD51 promotes homology donor search and base pairing. (**e**) Cell cycle dependency of DNA repair pathways: NHEJ is active through all cell cycle phases. Pathways requiring end resection are mainly active in the S-G2 phase.

The work of Maria Jasin’s group and collaborators indicated for the first time in 1994 that HDR is a major DSB repair pathway in mammalian cells, paving the way to the utilization of rare DNA-cutters, like CRISPR/Cas9, to promote HDR in mammalian cells [3]. Subsequent studies have also exploited NHEJ to promote loss of function editing by indels and integration at a DSB with rare DNA-cutters [4]. CRISPR mediated HDR is currently the most utilized method to facilitate targeted gene integration. However, the low efficiency of HDR in most eukaryotic cells is a major limitation. The activity of different DNA repair pathways at the DSB results in mixed editing outcomes. The deletions or insertions from NHEJ or a-EJ repair are mostly undesired in particular for therapeutically gene editing approaches. Finding ways to increase HDR efficiency, therefore, is a major goal in CRISPR genome editing research. This review describes recent approaches that have been made to improve HDR efficiency by small molecules. To set the stage main DSB repair pathways in mammalian cells will be introduced together with the key factors involved (Figure 1). A thorough depiction of DSB repair pathways is beyond the scope of this review, and for a more comprehensive overview, we recommend the review by Scully et al. (2019) [5].

## 2. Non-Homologous End-Joining Repair of Double-Strand Breaks

NHEJ is the predominant pathway to repair DSBs in mammalian cells. Broken ends can be repaired using various forms of end processing. In the initial recognition step, Ku70 and Ku80 bind sequence independently to lose DNA ends and form the Ku heterodimer. The complex forms a ring-shape, which keeps the free DNA ends in proximity of each other [6] and prevents end resection. The Ku complex recruits the DNA-Protein Kinase catalytical subunit (DNA-PKcs), which belongs to the PI3-Kinase related Protein Kinases (PI3KK) family. DNA-PKcs auto-phosphorylates and phosphorylates factors involved in NHEJ [7,8,9]. Autophosphorylation is required to make the DNA ends more accessible to repair enzymes to facilitate end ligation [10]. If needed to facilitate ligation, the nuclease Artemis processes incompatible ends [11]. Nucleotides are added in a template-dependent or -independent manner by the polymerases Polymerase Mu (POLM) and Polymerase Lambda (POLL) [12]. Compatible DNA ends are ligated by Ligase IV (LIG4), X-Ray Repair Cross-Complementing protein 4–XRCC4-like Factor (XRCC4-XLF) complex [13,14]. NHEJ is commonly believed to introduce mutations such as small insertions and deletions. However, it was shown that Ku-dependent end repair is in most cases precise [15]. A review from 2014 [16] emphasizes that the DNA end structure and amount of end processing needed for repairing the DSB, determine the precision of NHEJ. Brinkman and colleagues suggest, that the precise NHEJ-mediated repair of naturally occurring DSBs, is not exemplary for Cas9-induced DSBs [17] (Figure 1b).

## 3. Homology Directed Repair of Double-Strand Breaks

In contrast to NHEJ, HDR is inherently precise because it involves a homologous template to repair the DSB. In dividing cells, the sister chromatid serves as the perfect undamaged repair template. HDR requires extensive 5′–3′ end resection, resulting in single-stranded DNA (ssDNA) overhangs. End resection is initiated by CtBP-interacting protein (CtIP), which activates the MRN complex [18]. The MRN complex is formed by Meiotic Recombination 11 (MRE11), a 5′–3′exonuclease and 3′-5′endonuclease, RAD50, an ABC ATPase, and Nijmegen breakage syndrome 1 (NBS1). NBS1 interacts with Ataxia–Telangiectasia Mutated (ATM) or ATM- and Rad3-related (ATR) protein kinases. ATM and ATR are both phosphorylating proteins involved in cell cycle regulation and DNA repair. Like DNA-PKcs, they belong to the PI3KK family. ATM senses mainly DSBs, while ATR senses single-stranded DNA [19]. The initial end resection through MRN is followed by an extensive end resection through Exonuclease 1 (EXO1) or Bloom Helicase and DNA2 Helicase/Nuclease (BLM-DNA2) and coating of 3′ ssDNA with Replication Protein A (RPA) [20]. These processes are thereafter followed by RPA replacement with RAD51 loading through Breast Cancer Type 2 (BRCA2) with the help of Partner and Localizer of BRCA2 (PALB2) and Breast Cancer Type 1 (BRCA1). Furthermore, RAD52 has been shown to support RAD51 loading in an early phase of DSB repair [21]. The recombinase RAD51 together with the ssDNA nucleoprotein filament enables homology donor search and base-pairing [22]. The HDR process is finalized through either a mechanism called SDSA (Synthesis-dependent Strand Annealing) or DSBR (Double-Strand Break Repair Pathway), including the model of dHJ (double-Holliday Junction) intermediate [23] (Figure 1d).

## 4. Alternative End-Joining Repair of Double-Strand Breaks

Alternative End-Joining, also known as Microhomology-mediated end joining (MMEJ), shares characteristics of both the NHEJ and HDR pathway. The process is independent of Ku proteins as well as homologous repair templates but makes use of short homology regions in the genome (micro-homologies) to anneal strands. It requires short end resection by the MRN complex and CtIP [24]. To avoid extensive end resection Poly [ADP-ribose] Polymerase 1 (PARP1) interaction with the MRN complex plays an important role in a-EJ [25]. Microhomologies of 5–20 bp in the resulting 3′ssDNA anneal to each other. Resulting flaps are removed by X-Ray Repair Cross Complementing 1 (XLF-XRCC1) or Flap Endonuclease 1 (FEN1) [26]. DNA Polymerase Theta (POLQ) performs the filling of gaps [27] before the ends are ligated through Ligase I (LIG1) or the complex of Ligase III alpha (LIG3A)/XRCC1 [28]. Repair of DSB via a-EJ characteristically results in deletions. It should be noted that the intrinsically error-prone a-EJ pathway has been reported to show an only minor contribution to DSB repair in somatic cells and to be more relevant in cancer cells with defects in DNA repair pathways. Consequently, a-EJ was initially considered as a back-up to replace non-functional NHEJ or HDR processes. Currently, the contribution of a-EJ in cells with no deficiency in NHEJ or HDR is under investigation [29] (Figure 1c).

## 5. Double-Strand Break Repair Pathway Choice is Influenced by Cell Cycle Stage

The cell cycle stage is tightly linked to the regulation of end resection and thereby has an important role in the DSB repair pathway choice. NHEJ minimizes the processing required to join ends and is active throughout the cell cycle. HDR and a-EJ are initiated by end resection and are mainly active in the S and G2 phases as they depend on Cyclin-dependent Kinase (CDK) phosphorylation of downstream enzymes [24]. In the G1 phase, major mechanisms of end resection are suppressed. The two factors, BRCA1 and p53-Binding Protein 1 (53BP1), regulate the balance between NHEJ and HDR pathways throughout the cell cycle. In the G1 phase, phosphorylated 53BP1 binds to the DSB, prevents end resection and promotes NHEJ. Furthermore, during G1 phase BRCA1-dependent recruitment of PALB2 and BRCA2, and consequently the HDR machinery, the DSB is anticipated in a 53BP1 independent manner [30]. During the S/G2 phases, BRCA1 antagonizes the 53BP1 reaction and enables end resection, thus paving the way for HDR and a-EJ [31] (Figure 1e).

## 6. Small Molecules to Improve Precise Genome Editing

Targeted gene modification often suffers from low efficiency. HDR and NHEJ are competitive repair processes and different approaches to shift the balance towards HDR have been tested. In 2008, Beumer and colleagues showed that repair of zinc-finger induced DSB is channeled towards HDR in *Drosophila melanogaster* lines lacking Ligase IV, an important NHEJ factor [32]. As a result, down-regulation of NHEJ by gene knock-out [33], gene-silencing with siRNA [34] or shRNA [35] as well as low molecular weight compounds targeting involved molecules have been tested to indirectly increase HDR efficiency. Directly increasing HDR has been sought by overexpression of key HDR molecules [36], the fusion of them to Cas9 [37], or chemical enhancers of their activity. This review focuses on low molecular weight compounds to improve precise genome editing. Advantages of pharmacologically channeling DSB repair pathway choice to HDR include easy application to cell lines, reversibility, and fast mode of action. However, the availability of selective and potent inhibitors is limited and an appropriate candidate for the desired target might not be commercially available. Broadly used inhibitors show variable specificity, with some of them also inhibiting unintended targets [38]. Unwanted side-effects can be kept at a minimum by using the lowest possible concentration [39]. Subtle changes to the experimental design, such as donor design or treatment time-frame, can have an impact on the results. Therefore, confirmation of results and complementation of insights by independent studies is especially important for small molecule validation. Finally, pharmacological inhibition of target molecules is often more time- and labor- efficient than genetic knockout [39]. In the following NHEJ inhibitors will be introduced first, followed by direct enhancers of the HDR pathway. Cell-cycle modulators are discussed thereafter, before finally addressing inhibitors with unknown mechanisms. Small molecules addressed in this review are summarized in Table 1. They are shown with their functional target and observed effects in cell lines and animal models. It is further distinguished between single strand (ss) and double-strand (ds) donors, as it has been shown that repair by single-strand donors involves a different set of repair factors than repair by double-strand donors [40,41].

## 7. Increase of Homology Directed Repair by Inhibiting Non-Homologous End-Joining

HDR and NHEJ are competitive repair processes in response to DNA DSBs. It was, therefore, speculated that inhibition of NHEJ might shift the repair pathway choice towards HDR.

### 7.1. 53BP1

53BP1 recognizes ubiquitylated H2A at DSBs [65], prevents end resection, and thereby promotes NHEJ [31]. Thus, inhibition of 53BP1 might enable end resection and increase HDR rates. Canny and colleagues screened a library of ubiquitin variants, aiming to identify a protein that binds 53BP1 and prevents the interaction of 53BP1 with ubiquitylated histones at DSBs. The most promising ubiquitin variant, i53, was subsequently tested for its use in CRISPR applications. i53 expression in combination with a double-strand donor increased CRISPR induced insertion of a fluorescent marker into U2Os cells by nearly 2-fold. Expression of i53 in 53BP1^−/−^ cells did not further increase effects, demonstrating i53 to act via 53BP1 inhibition. In HEK293T cells, K562 cells and mouse embryo fibroblasts i53 increased the insertion of fluorescent reporters from double-strand donors into different gene loci by 1.3-fold, 1.8-fold, and 2.3-fold respectively. It was further shown that i53 expression increased HDR efficiency with single-strand donors for different target genes in several cell lines [42]. Paulsen and colleagues found that ectopic expression of a dominant-negative 53BP1 variant together with a single-stranded oligodeoxynucleotide (ssODN) donor significantly increased HDR efficiency in 3 out of 4 tested endogenous loci [66]. The fusion of dominant-negative 53BP1 to Cas9 increased HDR at different gene loci in HEK293T and hematopoietic cell lines on average by 2- to 3-fold [67]. These results thus suggest that inhibiting the binding of 53BP1 to DSBs could favor HDR repair. No low molecular weight compound, inhibiting 53BP1, has been described up to now.

### 7.2. Ku70/Ku80

Following a DSB, NHEJ is initiated by the binding of the Ku70/Ku80 heteroduplex to the DNA. Silencing of Ku70 or Ku80 by small-hairpin RNAs (shRNAs) was found to decrease NHEJ and increase HDR in transgenic HEK cells in response to CRISPR/Cas9 induced DSB [35]. This effect, however, might be a gene or cell type-specific or very dependent on the timing of the silencing, since siRNA knockdown of Ku70 in mouse ES cells showed no significant effect on HDR [34].

In 2016, Weterings and colleagues developed the first specific inhibitor of Ku70/Ku80 heterodimers. The chemical compound STL127705 inhibits the interaction between the Ku proteins and the DNA as well as Ku-dependent PKcs activation, both in vitro and in vivo [43]. There is currently no report where the effect of this inhibitor on the efficiency of HDR mediated genome editing has been evaluated.

### 7.3. DNA-PKcs

The PI3KKs, ATM, ATR, and DNA-PK, play a central role in the DNA damage response by phosphorylating various DNA repair factors during NHEJ. Knockdown of DNA-PKcs or its inhibition by the small molecule inhibitors NU7441 and KU-0060648 were found to reduce NHEJ repair and increase HDR 2- to 4-fold in HEK293T cells using a plasmid HDR donor or a ssODN donor [44].

The DNA-PKcs inhibitor NU7026 increased ssODN integration efficiency up to 1.6-fold for Cas9 editing in human induced pluripotent stem cells (hiPSC). Inhibitor treatment combined with a two double nick approach further increased targeted integration by up to 2.5-fold compared to untreated cells. Combining NU7026 (20 μM), Trichostatin A (0.01 μM), a histone deacetylase inhibitor, MLN4924 (0.5 μM), inhibiting neddylation of CtIP, and NSC15520 (5 μM), an inhibitor of the protein-protein interaction between the cell cycle checkpoint control proteins p53 or Rad9 and RPA70, in genome editing applications with Cas9 nickases increased KI efficiencies further [46]. A single treatment with NU7026 or combinatorial treatment with all four inhibitors showed an HDR promoting effect for Cpf1 induced DSBs. Except for NU7026, no other inhibitor increased HDR repair of Cas9 induced DSBs. In contrast to Cas9 Cpf1 produces staggered ends, which might explain potential differences. CRISPR/Cpf1 editing was also tested in two immortalized cell lines and two primary cell lines. NU7026 was the only inhibitor that showed a consistent increase in HDR efficiency [46].

Despite the successful increase of KI events using DNA-PKcs inhibitors, developing a selective and potent DNA-PK inhibitor has been a challenge in cancer therapy, due to sequence homologies between ATM, ATR, and DNA-PK [68]. Older generations of DNA-PKcs inhibitors such as NU7026, NU7441 or KU-0060648 show less selectivity against ATM and ATR, and/or are less potent compared to M3814, a new generation of DNA-PKcs inhibitor. In a follow-up study, Riesenberg and colleagues applied M3814 during genome editing. They were able to boost the effect of NU7026 more than 2-fold and showed a 4.5-fold increase of HDR in K562 cells. Additionally, they demonstrated the importance of DNA-PKcs activity for the pathway choice by involving cells with a kinase-deficient mutant of DNA-PKcs. In these cells a strong increase of HDR to NHEJ repair ratio was observed in 14 different genes in response to CRISPR/Cas9 introduced DSBs [33].

The use of DNA-PKcs inhibitors did not show an HDR increasing effect in mouse embryonic stem cells or mouse zygotes [34,47,48] and only a slight increase of HDR in a locus dependent manner using NU7441 in hiPSC [45]. This indicates effects to be highly dependent on cell-line and locus.

In 2018 a systematic screen of 600 inhibitors to increase Cpf1 mediated knock-in rates in hiPSC revealed compounds enhancing HDR efficiency by co-electroporation of the donor, guide, and Cpf1 plasmid. VE-822, an ATR inhibitor, was found to increase targeted integration with an optimal concentration of 1 μM. Further validation showed the inhibitor to increase HDR efficiency by 3-fold in combination with an ssODN donor and 5.9-fold with a plasmid donor. The efficiency could be further increased by combining the ATR inhibitor with a specific inhibitor of checkpoint kinase CHEK1 identified in the same screen [49].

### 7.4. DNA Ligase IV

An essential step during NHEJ repair is the ligation of the DSB ends, with Ligase IV being the key enzyme [13,14]. Inhibiting Ligase IV could thus decrease NHEJ and thereby also increase the HDR/NHEJ ratio. Ligase IV deficient *Drosophila melanogaster* lines display increased HDR efficiency for zinc-finger induced DSBs, supporting this hypothesis [32,69].

SCR7 is an inhibitor of Ligase IV, and to a lesser extent also Ligase III, which was first described as a potential cancer drug. It was hypothesized that inhibiting Ligase IV would inhibit NHEJ and thereby result in an increased number of unrepaired DSBs, which might cause increased sensitivity of cancer cells to radio- and chemotherapy. The inhibitor SCR7 was developed in silico to specifically bind Ligase IV and was demonstrated to interfere with the binding of Ligase IV to Ku-bound DNA fragments in a cell-free system and several cancer cell lines. In HeLa cells, an extrachromosomal fluorescent reporter system detected decreased NHEJ efficiencies after SCR7 treatment. In HeLa and MCF7 cells inhibitor treatment led to an increased number of γ-H2AX foci, indicative of an increased number of unrepaired DSBs. Importantly, cytotoxicity varied among different cancer cell lines, with sensitivity being particularly high in an HDR deficient cell-line [50]. This suggests that increased HDR could partially compensate for impaired NHEJ repair.

Maruyama and colleagues revealed that SCR7 facilitates HDR during CRISPR editing of cultured cells or mice. SCR7 increased the HDR mediated insertion rates of smaller fragments in A549 lung carcinoma cells and the melanoma line MelJuSo up to 19-fold and of a larger reporter construct into a dendritic cell line by approximately 13-fold. While melanoma cells showed a dose-dependent increase in HDR, 0.01 μM SCR7 was more effective than higher concentrations in epithelial A549 cells. Interestingly, SCR7 did not decrease the total number of mutations but caused a shift from NHEJ deletions to HDR insertions. The size distribution of insertions and deletions was not altered by SCR7 [51].

The insertion of a targeting construct into mouse zygotes was significantly increased by the co-microinjection of SCR7. Notably, neither viability of zygotes nor the number of live off-spring was impaired by SCR7 treatment [51], although knockout of Ligase IV in mice showed late embryonic lethality [70]. As in cell lines, the total number of genetic alterations was hardly changed, but deletions were reduced and insertions increased. Furthermore, SCR7 increased HDR efficiency in combination with either a single strand or double strand targeting donor [51].

Singh and colleagues confirmed the HDR promoting effect of SCR7 during CRISPR genome editing of mouse embryos. In contrast to the previous report, they did not include SCR7 in the microinjection mix but instead added it to the culture medium during microinjection. Short term culturing in 50 μM SCR7 overnight did not result in decreased viability of embryos, while prolonged culture showed a toxic effect. Inhibitor treatment increased the HDR efficiency approximately 10-fold and improved the HDR/NHEJ ratio [52].

Effects on HDR efficiency in response to SCR7 or Ligase IV knockdown were tested in transgenic HEK293 cells. A fluorescent reporter system distinguished between HDR mediated repair and NHEJ induced frameshifts. Increased HDR efficiency for either was detected. In contrast to Maruyama, decreased NHEJ efficiency was observed [35]. Increased KI rates after SCR7 treatment were moreover reported for MCF-7 and HCT-116 cells [53], and in rat embryos [54].

While SCR7 was shown to increase HDR mediated integration of dsDNA into fetal porcine fibroblasts [55], the efficiency to introduce a point mutation by ssODN could not be increased [56]. Estimation of NHEJ and HDR by an extrachromosomal fluorescent reporter construct in H1 cells did not show a significant difference in HDR or NHEJ efficiency between SCR7 treated and non-treated controls [57]. Similarly, a small scale study performed on rabbit embryos neither showed effects on NHEJ nor HDR [58]. These results suggest that SCR7 is acting in a cell type or gene-specific manner and might not be useful as a general promoter of HDR during CRISPR genome editing.

## 8. Increase of Template-Directed Repair by Facilitation of the Homology Directed Repair Pathway

### RAD51

Another option to improve the efficiency of HDR mediated genome editing is to directly target molecules involved in HDR. The binding of helical RAD51 filaments to ssDNA is a crucial step in HDR. RAD51 overexpression had been reported to promote HDR in Chinese Hamster Ovary (CHO) fibroblasts [36] and human ES cells [64], while knock-out of RAD51 paralogues resulted in reduced HDR efficiency in chicken B-lymphocyte DT40 cells [71,72]. Jayathilaka and colleagues performed an in vitro screen with 10,000 small molecules to find compounds that modulate RAD51 binding to ssDNA. RS-1 was identified to enhance hRAD51 binding activity in excess of 2-fold and it was suggested that RS-1 might be useful to improve HDR-based targeted mutagenesis [59]. Indeed, the Dellaire group later reported that HDR mediated integration of a fluorescent reporter into HEK293A or U2OS cells was increased 3- to 6-fold after treatment with 10 μM RS-1. At the same time, indel rates were not altered after inhibitor treatment. It was further shown that the inhibitor can also be used to increase knock-in (KI) rates after double nicking of the DNA with Cas9 nickases (~4-fold). However, in another experiment in the same study, RS-1 resulted only in a moderate KI increase of about 0.3-fold [60]. The reason for this inconsistency was not discussed.

RS-1 treatment was also shown to increase nuclease mediated gene targeting of rabbit embryos. Using cultured embryos, the Zhang group demonstrated a significant increase of HDR at two different genetic loci after treatment with 7.5 μM RS-1. This suggests an HDR promoting effect independent of the target gene. Surprisingly though, a higher concentration of RS-1 (15 μM) did not increase HDR efficiency but increased blastocyst viability. Additional experiments included implantation and fetal development of the kits. Knock-in efficiencies were tested for two different genomic loci. In both cases, the number of embryos with successful KI was increased by ~3-fold, although the total number of successfully targeted kits was low. Notably, treatment with RS-1 did not reduce the viability of kits. No obvious alteration of NHEJ mediated indels was observed [58].

Very recently, HDR promoting effects of RS-1 have also been confirmed in bovine embryos. After microinjection of the CRISPR components and an ssDNA repair template, in vitro fertilized zygotes were incubated in 7.5 μM RS-1. KI rates were doubled compared to embryos not treated with RS-1 [61]. Another recent study, however, did not observe an effect with RS-1 [41].

Taken together, RS-1 displayed quite some variation in promoting HDR. More studies are needed to understand the underlying molecular reason. This knowledge might enable us to predict in which cell types or for which target genes RS-1 will have a beneficial effect on HDR efficiency.

## 9. Increase of Homology Directed Repair by Cell Cycle Synchronization

While NHEJ is possible during all phases of the cell cycle, HDR has been described to peak during S and G2 phases [73]. Performing CRISPR genome editing exclusively at S and G2 phase might thus increase HDR efficiency.

The Doudna group tried to achieve this goal with the help of cell cycle inhibitors, which stalled the cell cycle at different stages. Immediately after the release of the cell cycle block, cells were nucleofected with preassembled Cas9 ribonucleoprotein (RNP) complex. This restricts the editing timeframe to approximately 24 h after delivery, due to Cas9 degradation. Of four G1/S blockers tested, only aphidicolin showed an HDR promoting effect both in HEK293T cells and in neonatal fibroblasts. Three other G1/S inhibitors (mimosin, thymidine, hydroxy urea) increased HDR in neonatal fibroblasts but surprisingly decreased HDR in HEK293T. Unexpectedly, the G2/M inhibitor nocodazole displayed the strongest HDR promoting effect in HEK293T cells. As possible explanations, the authors suggest that timed-RNP delivery into a cell prior to division might effectively target two cells. Additionally, at the M-phase the nuclear membrane is broken down, which might facilitate RNP delivery. Nocodazole synchronization, however, did not increase HDR at the EMX1 gene in neonatal fibroblasts and human ES cells [62]. Off-target effects of the inhibitors might contribute to unexpected behavior of the cell cycle inhibitors. An HDR promoting effect by G2/M cell cycle inhibitors was confirmed in human stem cell lines where nocodazole and ABT-751 enhanced HDR CRISPR genome editing of different genes. HDR promoting effects were also detected for the repair of DSBs induced by CRISPR N. meningitides. Strikingly, pluripotent stem cells remained pluripotent during the editing process and could be differentiated into all three germ layers at a later time point. This demonstrates cell cycle synchronization to be a robust and easy tool to increase HDR in undifferentiated cell lines [57].

The Corn group performed a screen to identify regulators of HDR with a double-stranded DNA repair template. Using a reporter system for HDR driven conversion of Blue Fluorescent Protein (BFP) to Green Fluorescent Protein (GFP) expressed in K562 cells, they identified CDC7 as a repressor of HDR [41]. Since XL413 had previously been shown to specifically inhibit CDC7 [74] they then investigated whether this inhibitor might increase HDR efficiency. Indeed, the BFP-to-GFP reporter system revealed a significant increase in HDR by 1.4-fold and 1.8-fold with single-strand and double-strand donors respectively. Similar results were achieved for additional gene targets and the insertion of smaller substitutions as well as bigger transgenes. An HDR promoting effect for XL413 was also shown for different loci in T cells and HSPCs. Tests in eight additional cell lines revealed a cell-to-cell variation of inhibitor effect: Four cell lines showed varying effects with an increase of HDR at some gene loci. The remaining four cell lines never showed an increase in HDR by inhibitor treatment. Other inhibitors tested in parallel showed either no (RS-1, SCR7) or a variable effect (L755507, aphidicolin, hydroxy urea) in K562 cells. An increase in HDR by i53 treatment has been shown to depend on transfection efficiency. Functional analysis of XL413 effects on cell cycle suggests that arresting cells at early S-phase extends the S/G2/M phase. The inhibitor was thus validated as a new cell cycle regulator that increases HDR rates [41].

The influence of the cell cycle on HDR has been demonstrated by a genetically engineered Cas9-geminin fusion protein, which is degraded during the late M or G1 phase but highly expressed during S/G2/M phase of the cell cycle. HDR efficiencies could be increased by up to 87% compared to non-modified Cas9. Transient cell-cycle arrest by nocodazole during genome editing with the transfected Cas9-geminin fusion protein showed an additive effect and resulted in higher HDR levels than one condition alone [75].

## 10. Increase of Homology Directed Repair Efficiency by Inhibitors via Undetermined Mechanism

To identify activators of HDR, Yu et al. (2015) screened nearly 4000 small molecules with pharmacological activity for the increase of HDR mediated integration of GFP into the Nanog locus in murine ES cells. L755507, a β3-adrenergic receptor agonist, and brefeldin A, an inhibitor of the intracellular transport from the endoplasmic reticulum (ER) to Golgi, were found to increase HDR 3- and 2-fold, respectively. L755507 was tested in additional cell types and other targeting loci, for which also an improved targeting efficiency was observed. An even higher improvement was observed when a ssODN template was used. How these molecules exert their function on HDR is not clear. For L755507 a small decrease in NHEJ repair efficiency was detected [63].

Li and colleagues confirmed the effect of L755507 in fetal porcine cells and reported in the same study that resveratrol promotes HDR. Interestingly, L755507 and in particular resveratrol increased the expression of several HDR associated genes including RAD51. This correlated in the case of resveratrol with a strong dose-dependent cytotoxic effect and an increased percentage of cells in the S phase of the cell cycle. L755507 decreased expression of several NHEJ repair-related genes, but resveratrol increased their expression [55]. More research is needed to understand the mechanism underlying the HDR promoting effect of L755507, brefeldin A, and resveratrol.

In 2012 Takayama and colleagues hypothesized that loosening the chromatin structure at transcriptionally inactive sites might increase HDR efficiency during CRISPR editing. They screened an epigenetic library and identified valproic acid (VPA) as an HDR promoting compound in human ES cells. 24 h pre-treatment with VPA increased HDR efficiency at a transcriptionally active as well as an inactive site compared to non-treated control cells. It was further demonstrated that acetylation levels at H3K9, H3K27, and H4K16 were significantly increased at either targeting site [64]. Already earlier it had been reported that VPA is increasing the frequency of homologous recombination [76].

## 11. Conclusions

Increasing HDR efficiency in a simple, reproducible manner is one of the most important requirements to promote gene repair. Improved HDR efficiency will not only facilitate the use of CRISPR genome editing in therapeutic settings but will also facilitate the use of CRISPR in biomedical and drug discovery in general, from model generation to the study of disease variants.

Several small molecules have been described that promote HDR either directly or indirectly by interfering with several steps of NHEJ. However, up to now none of them has become part of the standard procedure of CRISPR genome editing. This is probably at least partially because the inhibitors show a considerable cell-to-cell variation of their effects and different efficiencies for specific genomic sites. The underlying molecular reasons are poorly understood. High throughput screens or testing of small molecules to enhance HDR are usually performed in immortalized cell lines, such as HEK293T. These transformed cell lines, modified to be easy to culture and manipulate, can differ amongst others in their DNA damage response, cell cycle control, or ploidy compared to primary cells. The effect of a selected small molecule should be evaluated for its functionality in the cell type of interest. Also, the effect of low molecular weight compounds on CRISPR-related off-target effects, such as off-target cutting, translocation, and large deletions, must be carefully assessed [77,78].

More detailed knowledge of the DSB repair pathways and the possible role of chromatin modifications in the regulation of genome editing will be instrumental to overcome the variation or to predict which inhibitors will work best in a given cell line for a given gene.

High-throughput screens might also be used to identify novel HDR promoting genes and small molecules. As chromatin packing affects the ratio of NHEJ to HDR [79], chromatin-modifying genes could be tested for their role in HDR/NHEJ pathway choice.

Broad screens have been carried out with pharmacologically active substances and HDR efficiency as a readout. However, a systematic analysis of druggable targets in the NHEJ and HDR pathways to enable screens for low molecular weight substances interfering or promoting specific protein-protein interactions is still lacking. Such focused screens, ideally also incorporating structural information on the target protein, could potentially result in more effective HDR promoters than currently available.

Moreover, genetic screens using genome-wide CRISPR-screens can reveal new targets to enhance gene integration. Once a new target has been identified small molecules can be systematically designed against the target by modifying their catalytic activity or protein-protein interactions. In addition, advances in the development of new modalities such as PROteolysis TArgeting Chimeras (ProTacs) will extend the number of druggable targets [80].

Off-target effects and toxicities could prevent widespread use of HDR promoting small molecules. This is particularly a problem if different inhibitors are combined in a cocktail. Still, combinatorial treatment with different HDR promoting small molecules might be a way to overcome cell type and gene specificities of individual inhibitors. Existing high-throughput systems could be used to test the effect of different inhibitor combinations and various concentrations.

Alternative strategies based on NHEJ, MMEJ, Prime Editing, or Transposon mediated KI, as well as Base Editing, may be more efficient in G1 resting cells due to the strict dependency between HDR and particular phases of the cell cycle [81,82,83,84,85]. Prime Editing and Base Editing directly fuse Cas- nickases to an effector protein (a DNA deaminase for Base Editing and a Reverse Transcriptase for Prime Editing). This can overcome the limitations of DSB repair pathway dependency. However, it is possible that small molecules interfering with DNA metabolism can further promote the efficiency of Prime and Base Editing. Taken together, promising small molecule activators of HDR have been described, but additional work is required to translate this knowledge into a standard protocol for the broad use in CRISPR genome editing.

## Figures and Tables

**Table 1:** Summary of small molecules described during this review with suggested targets and observed effects.

Small Molecule	Target	Observed Effects	Sources
i53	Prevents interaction of 53BP1 with ubiquitylated histones at DSBs	Increased HDR with ss and ds donor in several cell lines	[42]
STL127705	Inhibits interaction of Ku proteins with DNA and Ku-dependent PKcs activation	Not tested	[43]
NU7441	Inhibition of DNA-PKcs	Reduces NHEJ and increases HDR in HEK293T cells using ss or ds donors Minor increase in hiPSC with ds donor	[44,45]
KU-0060648	Inhibition of DNA-PKcs	Reduces NHEJ and increases HDR in HEK293T cells using ss or ds donors	[44]
NU7026	Inhibition of DNA-PKcs	Increased KI with ss donor after Cas9 induced DSB or double nicking or Cpf1 induced DSB in hiPSC No effect in mouse embryonic stem cells or in mouse zygotes with ds donor	[34,46,47,48]
M3814	Inhibition of DNA-PKcs	Increased KI in hiPSC and K562 cells with ss donor using Cas9 or Cpf1	[33]
VE-822	Inhibition of ATR	Increases HDR in hiPSC with ss or ds donor in combination with Cpf1	[49]
SCR7	Inhibitor of Ligase IV	Decreased NHEJ repair of an extrachromosomal reporter system in HeLa cells Increased HDR in several cell lines with ds donor or ss donor Increased HDR in mice with ss donor No HDR increase in rabbits with ds donor Increased HDR in rats with ds donor No HDR increase for an extrachromosomal reporter in H1 cells Inconsistent effects on HDR efficiency in fetal porcine fibroblasts	[35,50,51,52,53,54,55,56,57,58]
RS-1	Enhances RAD51 binding to ssDNA after end-resection	Increased HDR in cell lines and rabbit embryos with ds donor Increased HDR in bovine embryos with ss donor	[58,59,60,61]
Aphidicolin	G1/S blocker	HDR promoting effect in HEK293T and neonatal fibroblasts with ss donor	[62]
Mimosin, thymidine, hydroxy urea	G1/S blocker	Increased HDR in neonatal fibroblasts with ss donor Decreased HDR in HEK293T cells with ss donor	[62]
Nocodazole	G2/M blocker	HDR promoting effect in HEK293T cells with ss or ds donor No HDR increase in neonatal fibroblasts or human ESC using ss donor Increased HDR in hPSC with ds donor	[57,62]
ABT-751	G2/M blocker	Increased HDR in hPSC with ds donor	[57]
XL413	G1/S blocker	Increased HDR in K562 cells and T cells with ss or ds donor Increase in HSPCs with ss donor (ds not tested) Additionally tested cell lines showed either varying or no effects	[41]
L755507	β3-adrenergic receptor agonist	Increase in HDR in several cell lines with ss and ds donors	[55,63]
Brefeldin A	Inhibition of intracellular transport from ER to Golgi	Increase in HDR in mES cells with ds donor	[63]
Resveratrol	Broad range of biological activities	Increase of HDR in porcine fetal fibroblasts with ds donor	[55]
VPA	HDAC inhibitor	Increase of HDR in human ESC with ds donor	[64]

Shown are small molecules that are suggested to directly increase HDR (RS-1), indirectly increasing HDR via inhibition of NHEJ (i53, STL127705, NU7441, KU-0060648, NU7026, M3814, VE-822, SCR7), regulation of cell cycle (aphidicolin, mimosin, thymidine, hydroxy urea, nocodazole, ABT-751, XL413) or via undetermined pathways (L755507, brefeldin A, resveratrol, VPA). Functional aspects of inhibition are described under targets. Observed effects summarize results for treatment with single inhibitors as reviewed from the specified sources. ATR = ATM-Rad3- related, DNA-PKcs = DNA-Protein Kinase catalytical subunit, ds = double strand, DSB = Double-Strand Break, ER = endoplasmic reticulum, ESC = embryonic stem cells, HDAC = histone deacetylases, HDR = Homology Directed Repair, hiPSC = human induced pluripotent stem cells, hPSC = human pluripotent stem cells, HSPCs = hematopoietic stem and progenitor cells, mES cells = mouse embryonic stem cells, NHEJ = Non-Homologous End-Joining, ss = single strand, ssDNA = single-stranded DNA, VPA = valproic acid, 53BP1 = p53-Binding Protein 1.
